# Potentiation of Glutamatergic Synaptic Transmission Onto Dorsal Raphe Serotonergic Neurons in the Valproic Acid Model of Autism

**DOI:** 10.3389/fphar.2018.01185

**Published:** 2018-10-16

**Authors:** Ruixiang Wang, Kathryn Hausknecht, Roh-Yu Shen, Samir Haj-Dahmane

**Affiliations:** ^1^Research Institute on Addictions, University at Buffalo, The State University of New York, Buffalo, NY, United States; ^2^Department of Psychology, University at Buffalo, The State University of New York, Buffalo, NY, United States; ^3^Department of Pharmacology and Toxicology, The Jacob School of Medicine and Biomedical Sciences, University at Buffalo, The State University of New York, Buffalo, NY, United States; ^4^Neuroscience Program, University at Buffalo, The State University of New York, Buffalo, NY, United States

**Keywords:** autism, valproic acid, anxiety, dorsal raphe nucleus, 5-HT, glutamatergic synapse, E/I imbalance, hyperserotonemia

## Abstract

Autism spectrum disorder (ASD) is characterized by social and communicative impairments and increased repetitive behaviors. These symptoms are often comorbid with increased anxiety. Prenatal exposure to valproic acid (VPA), an anti-seizure and mood stabilizer medication, is a major environmental risk factor of ASD. Given the important role of the serotonergic (5-HT) system in anxiety, we examined the impact of prenatal VPA exposure on the function of dorsal raphe nucleus (DRn) 5-HT neurons. We found that male rats prenatally exposed to VPA exhibited increased anxiety-like behaviors revealed by a decreased time spent on the open arms of the elevated plus maze. Prenatal VPA exposed rats also exhibited a stereotypic behavior as indicated by excessive self-grooming in a novel environment. These behavioral phenotypes were associated with increased electrical activity of putative DRn 5-HT neurons recorded *in vitro*. Examination of underlying mechanisms revealed that prenatal VPA exposure increased excitation/inhibition ratio in synapses onto these neurons. The effect was mainly mediated by enhanced glutamate but not GABA release. We found reduced paired-pulse ratio (PPR) of evoked excitatory postsynaptic currents (EPSCs) and increased frequency but not amplitude of miniature EPSCs in VPA exposed rats. On the other hand, presynaptic GABA release did not change in VPA exposed rats, as the PPR of evoked inhibitory postsynaptic currents was unaltered. Furthermore, the spike-timing-dependent long-term potentiation at the glutamatergic synapses was occluded, indicating glutamatergic synaptic transmission is maximized. Lastly, VPA exposure did not alter the intrinsic membrane properties of DRn 5-HT neurons. Taken together, these results indicate that prenatal VPA exposure profoundly enhances glutamatergic synaptic transmission in the DRn and increases spontaneous firing in DRn 5-HT neurons, which could lead to increased serotonergic tone and underlie the increased anxiety and stereotypy after prenatal VPA exposure.

## Introduction

Autism spectrum disorder (ASD) is a prevalent and serious neurodevelopmental disorder, which is characterized by social impairments, deficits in communication skills, restricted social interests, and repetitive behaviors (American Psychiatric Association, 2013). Moreover, ASD is often comorbid with other mental health problems, including excessive anxiety and fear (White et al., 2009). Work conducted over the last decades has shown that ASD is a highly heritable disorder and identified approximately 800 mutated genes as potent risk factors (Abrahams and Geschwind, 2008; Abrahams et al., 2013; Tick et al., 2016). These genes control synaptogenesis and the function of several neurotransmitter systems, including the serotonergic (5-HT) system. Indeed, early studies have reported hyperserotonemia, i.e., elevated whole blood serotonin levels, as the first common biomarker found in ASD patients (Schain and Freedman, 1961; Muller et al., 2016). A meta-analysis showed elevated 5-HT blood levels in 28.3% of autistic individuals (Gabriele et al., 2014). Hyperserotonemia is also associated with increased repetitive and anxiety-like behaviors (Cook et al., 1994; Muller et al., 2016). Moreover, 5-HT-related gene variants have been linked to ASD. For example, mutations of tryptophan hydroxylase-2 and the short allelic variant of 5-HTTLPR, a polymorphic region on the 5-HT transporter (5-HTT) gene SLC6A4, have been identified as genetic risk factors for ASD (Devlin et al., 2005; Huang and Santangelo, 2008; Arieff et al., 2010). The role of the 5-HT system in ASD is also supported by animal studies showing that mutation of the 5-HTT gene (Muller et al., 2016) or deletion of monoamine oxidase type A (MAOA) leads to ASD-like phenotypes (Gobbi et al., 2005). Collectively, these human and animal studies support a critical role of 5-HT neurotransmission in ASD, which may mediate the increased anxiety phenotype.

In addition to the genetic factors, growing evidence indicates that prenatal and early postnatal environmental factors contribute to ASD (Landrigan, 2010; Grabrucker, 2013; Ornoy et al., 2015). Indeed, prenatal and postnatal exposure to various teratogens, such as valproic acid (VPA), has been shown to profoundly increase the risk for ASD. In humans, exposure to VPA during the first trimester of the gestational period enhances the incidence of ASD in the offspring (Christensen et al., 2013). Similarly, animal studies have consistently reported that prenatally (on embryonic days 11–12.5) VPA-exposed rats exhibit neurological and behavioral deficits similar to human ASD. Therefore, prenatal VPA exposure in rats has been used as a valid animal model to elucidate the neuronal mechanisms mediating behavioral deficits in ASD (Schneider and Przewłocki, 2005; Roullet et al., 2013).

Despite the role of 5-HT neurotransmission in the pathophysiology of ASD, it remains unknown whether the function of dorsal raphe nucleus (DRn) 5-HT neurons, a major source of 5-HT in the brain (Dahlström and Fuxe, 1964; Descarries et al., 1982), is altered in an animal model of ASD. In this study, we examined the function of putative DRn 5-HT neurons in the VPA model of ASD. We found that prenatal VPA exposure increased anxiety-like behaviors in male rats. These behavioral effects were associated with activation of DRn 5-HT neurons and potentiation of glutamatergic synaptic transmission onto DRn 5-HT neurons. Together, these results help unravel previously unsuspected effects of prenatal VPA exposure on the function of glutamatergic synapses onto DRn 5-HT neurons.

## Materials and Methods

### Animals and Prenatal VPA Exposure

Rats were bred in house. Briefly, Long Evans male and virgin female breeders (Envigo, Indianapolis, IN, United States) were housed in pairs in breeding cages, food and water *ad lib*. The colony room was maintained with a 12 h/12 h light/dark cycle (light on at 7:00 AM – 7:00 PM), temperature at 20 – 26°C and humidity at 40 – 60%. Rat droppings were carefully monitored on a daily basis until copulatory vaginal plugs were unequivocally identified (embryonic day 1). Then pregnant females were singly housed in standard plastic cages. VPA (sodium salt) was administered (400 mg/kg, s.c., dissolved in saline at 100 mg/ml; vehicle: 4 ml/kg saline) on embryonic day 12.5. The VPA dose and exposure window were similar to those used in other studies (Roullet et al., 2013; Nicolini and Fahnestock, 2018). The pups were weaned on postnatal day (PD) 21, and then same-sex rats were housed in pairs in standard plastic cages until the completion of the experiments. Only male rats were used in the present study. Electrophysiological recordings were conducted at the age of 6–8 weeks, and behavioral tests were performed at the age of 8 weeks. All the procedures were carried out with approved protocols from the University at Buffalo Institutional Animal care and Use Committee and were in accordance with the National Institutes of Health guidelines for the Care and Use of Laboratory Animals.

### Self-Grooming Test

The self-grooming test was performed in an open field plastic box (55 cm × 38 cm × 42 cm), during the dark phase of the light/dark cycle. Activity of rats was recorded by an infrared digital camera mounted above the box and connected to a computer. The rat was allowed to habituate to the dimly lit testing room for 15 min, and then was placed in the center of the open field. Recording lasted for 5 min, and duration of self-grooming was measured by manually coding the video clip by individuals blind to the experimental groups. Longer duration of self-grooming in a novel environment indicates greater anxiety in the rat (Gispen and Isaacson, 1981; Kalueff and Tuohimaa, 2005).

### Elevated Plus Maze (EPM) Test

The EPM test was conducted during the dark phase. The wood maze was elevated 50.8 cm from the floor, with two open arms and two closed arms (50.8 cm × 10.15 cm for each of the 4 arms). The closed arms were fenced by 50.8 cm-tall transparent Plexiglas walls. Activity of rats was recorded by an infrared digital camera mounted above the maze and connected to a computer. Animals were allowed to habituate to the dimly lit testing room for 15 min prior to the test, and then placed on the center of the maze with the head facing an open arm. Each recording lasted for 5 min, and the video clip was manually coded later by individuals blind to the experimental groups. The rat’s entries into and durations of stay/travel on the open and closed arms were analyzed. The numbers of entries indicate the animal’s locomotion level, more entries suggesting higher locomotor activity. The time spent on the open arms indicates the magnitude of anxiety. A shorter stay on the open arms is indicative of augmented anxiety (Hogg, 1996; Rodgers and Dalvi, 1997).

### Brain Slice Preparation

Brain slices containing the DRn were prepared using previously described procedures (Haj-Dahmane, 2001). In brief, rats were anesthetized with 0.5% isoflurane and killed by decapitation. A block of the brainstem area containing the DRn was isolated and coronal slices (300–350 μm) were cut using a vibratome (Lancer series 1000, Leica Biosystem, St Louis, MO, United States) in ice-cold modified artificial cerebrospinal fluid (ACSF) of the following composition (in mM): 110 choline-Cl; 2.5 KCl; 0.5 CaCl_2_; 7 MgSO_4_; 1.25 NaH_2_PO_4_; 26.2 NaHCO_3_; 11.6 sodium L-ascorbate; 3.1 sodium pyruvate; and 25 glucose, equilibrated with 95% O_2_/5% CO_2_. Slices were incubated for 45 min at 35°C and then at room temperature for at least 1 h in a holding chamber containing standard ACSF (in mM): 119 NaCl; 2.5 KCl; 2.5 CaCl_2_; 1.3 MgSO_4_; 1 NaH_2_PO_4_; 26.2 NaHCO_3_; and 11 glucose, continuously bubbled with a mixture of 95% O_2_/5% CO_2_. Following recovery, slices were transferred to a recording chamber (Warner Instruments, Hamden, CT, United States) mounted on a fixed upright microscope (Olympus BX51, Olympus Co., Tokyo, Japan) and continuously perfused (2–3 ml/min) with ACSF, saturated with 95% O_2_/5% CO_2_ and heated to 30 ± 1°C using a solution heater (Warner Instruments).

### Electrophysiological Recordings

The DRn neurons were visualized using the Olympus BX51 microscope equipped with a 40x water-immersion lens, differential interference contrast, and infrared optical filter. Somatic whole cell recordings were obtained from putative DRn 5-HT neurons with patch electrodes (3–5 mΩ) filled with a solution containing (in mM): 120 potassium gluconate; 10 KCl; 10 Na_2_-phosphocreatine; 10 HEPES; 1 MgCl_2_; 1 EGTA; 2 Na_2_-ATP; 0.25 Na-GTP (pH 7.3, osmolality 280–290 mOsm). The DRn 5-HT neurons were identified by their distinct electrical properties, which include slow firing activity induced by supra-threshold membrane depolarization, large after-hyperpolarization and membrane hyperpolarization induced by 5-HT_1A_ receptor agonist as previously described (Haj-Dahmane, 2001; Geddes et al., 2015). For the experiments assessing excitation/inhibition (E/I) ratio, whole cell recordings were performed with intracellular solution containing (in mM) : 120 cesium methanesulfonate; 10 KCl; 10 Na2-phosphocreatine; 10 HEPES; 1 MgCl2; 1 EGTA; 2 Na_2_-ATP; 0.25 Na-GTP (pH 7.3, osmolality 280–290 mOsm).

All recordings were performed from putative 5-HT neurons located in the dorsomedial subdivision of the DRn. Excitatory postsynaptic currents (EPSCs) or inhibitory postsynaptic currents (IPSCs) were evoked with single square-pulses (duration = 100–200 μs) delivered at 0.1 Hz with patch pipettes (2–3 mΩ) filled with standard ACSF and placed (50–100 μm) dorsolateral to the recording sites. In some experiments, to assess changes in paired-pulse ratio (PPR), pairs of EPSCs or IPSCs were evoked with an inter-stimulus interval (ISI) of 30–150 ms. The intensity of the stimulus was adjusted to evoke 75% of the maximal amplitude of EPSCs/IPSCs. The EPSCs were recorded from neurons voltage clamped at -70 mV in the presence of gamma-aminobutyric acid type A (GABA_A_) and glycine receptor antagonists picrotoxin (100 μM) and strychnine (20 μM), respectively. GABA_A_ receptor-mediated IPSCs were recorded from neurons voltage-clamped at 0 mV in the presence of N-methyl-D-aspartic acid (NMDA) and AMPA receptor antagonists D-AP5 (50 μM) and DNQX (20 μM), respectively. To examine miniature action potential-independent EPSCs (mEPSCs), tetrodotoxin (1 μM) was added in the bath and mEPSCs were recorded for 150 s. Membrane currents were amplified with an Axoclamp 2B or Multiclamp 700B amplifier (Molecular Devices, Union City, CA, United States). Membrane currents were filtered at 3 kHz, digitized at 20 kHz with Digidata 1440A, and acquired using the pClamp 10 software (Molecular Devices). The cell input resistance and access resistance (10–20 mΩ) were monitored throughout the experiments using 5 mV hyperpolarizing voltage steps (500 ms duration). Recordings were discarded when the input and series resistance changed by more than 10–20%.

To examine whether glutamatergic synapses onto DRn 5-HT neurons exhibit activity-dependent plasticity, we used a spike-timing-dependent long-term potentiation (tLTP) induction protocol that consisted of pairing a train of five bursts of presynaptic stimulation with back-propagating action potentials (bAPs) delivered at 5 Hz. Each burst was composed of three presynaptic stimuli (50 Hz) paired with three bAPs (50 Hz) with a delay of + 5 to 10 ms. Action potentials were evoked by injection of depolarizing somatic current (1.5–2 nA, 2 ms duration) in the current clamp mode. After obtaining a stable recording of EPSCs for at least 10 min, the recordings of DRn 5-HT neurons were switched to the current clamp mode and a total of 20 trains were administered at 0.1 Hz.

### Data Analyses

The dependent variables of the behavioral tests were described above. For the electrophysiological recordings, the amplitudes of EPSCs and IPSCs were analyzed using Clampfit 10.2 software (Molecular Devices) and determined by measuring the average current during a 2 ms time window at the peak of each EPSC/IPSC, subtracted from the baseline current determined during a 5 ms time window before the stimulus artifact. The E/I ratio was defined as the ratio of the amplitudes of EPSCs and IPSCs obtained in the same neuron. The PPRs (EPSC2/EPSC1 or IPSC2/IPSC1) were averaged for at least 60 trials. For the analysis of tLTP, EPSC amplitudes were normalized to the mean baseline amplitude recorded for at least 10 min before the administration of the pairing protocol. Statistical analyses were performed, using Student’s *t* tests, analysis of variance (ANOVA), Kolmogorov–Smirnov tests (K–S tests), or Chi-square tests, with software SAS 9.4 (SAS Institute, Cary, NC, United States) or Origin 8.0 (MicroCal Software Inc., Northampton, MA, United States). Pairwise comparisons after ANOVA were conducted using *post hoc* Tukey tests. Statistical significance was set at α = 0.05. The results in the text and figures are expressed as mean ± SEM.

### Chemicals

Most chemicals were obtained from Fisher Scientific (Pittsburgh, PA, United States). VPA sodium salt and 6,7-dinitroquinoxaline-2,3-dione (DNQX) were purchased from Sigma-Aldrich (Saint Louis, MO, United States). Picrotoxin, strychnine, and D-(-)-2-Amino-5-phosphonopentanic acid (D-AP5) were obtained from Tocris Biosciences (Minneapolis, MN, United States).

## Results

### Prenatal VPA Exposure Slightly Reduces Bodyweights on PD1 but Not Litter Sizes

Fourteen control and 23 VPA female rats were used in breeding, among which 13 control and 20 VPA exposed rats gave birth to live pups. A Chi-square test showed no significant group difference in proportion of litters with live pups. For number of live pups/litter, there was no significant group difference (Control = 9.54 ± 0.95; VPA = 7.45 ± 0.94), based on a two-way ANOVA (group: control vs. VPA; sex: male vs. female). In addition, prenatal VPA exposure resulted in slightly lower body weights on PD 1 in both male and female pups (Control Male: 7.05 ± 0.11 g; VPA Male: 6.47 ± 0.10 g; Control Female: 6.68 ± 0.11 g; VPA Female: 6.13 ± 0.11 g). A two-way nested ANOVA with litter as a nested variable produced a group main effect, *F*_1,246_ = 22.61, *p* < 0.001, a sex main effect, *F*_1,246_ = 9.83, *p* < 0.01, and a litter effect, *F*_23,246_ = 8.67, *p* < 0.001. Taken together, these results suggest that prenatal VPA treatment did not produce major teratogenic effects.

### Prenatal VPA Exposure Increases Stereotypy and Anxiety-Like Behaviors

**FIGURE 1:**
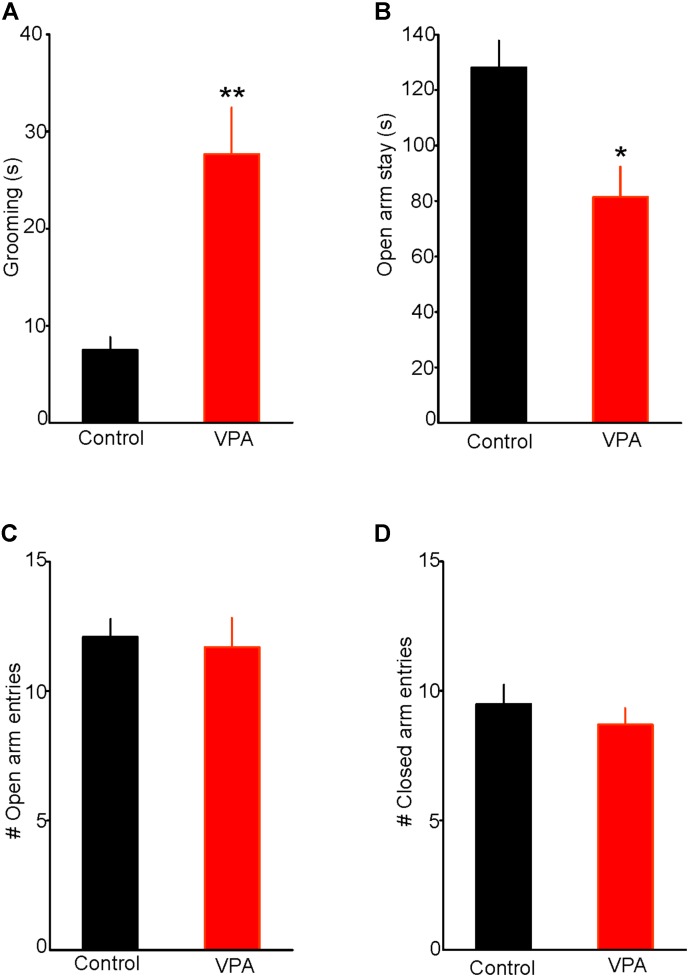
Prenatal valproic acid (VPA) exposure increases anxiety-like behaviors. **(A)** Effect of prenatal VPA exposure on self-grooming. Note the significant increase in duration of self-grooming in VPA rats, indicating augmented anxiety (Control: *n* = 7; VPA: *n* = 8, *p* < 0.01). **(B)** Effect of prenatal VPA exposure on the duration of open arm stay in the elevated plus maze. Note the significant reduction in open arm stay in VPA rats (Control: *n* = 10; VPA: *n* = 7, *p* < 0.05). **(C,D)** illustrate the effects of prenatal VPA exposure on the numbers of open and closed arm entries, respectively. Note that prenatal VPA exposure does not alter the numbers of entries into the open or closed arms (Control: *n* = 10; VPA: *n* = 7; *p* > 0.05 for both). Data are presented as Mean ± SEM. ^∗^*p* < 0.05; ^∗∗^*p* < 0.01.

Results from previous animal studies have shown that prenatal VPA exposure induces a robust ASD-like phenotype, including repetitive behaviors (Lewis et al., 2007; Olexová et al., 2016; Nicolini and Fahnestock, 2018). Consistent with this notion, we found that a single injection of VPA (400 mg/kg; s.c.) on embryonic day 12.5 significantly increased self-grooming, a measure of stereotypy associated with augmented anxiety. Indeed, as illustrated in **Figure 1A**, VPA exposed rats spent more time engaging in self-grooming in a novel environment, compared with control rats (Control = 7.5 ± 1.3 s, *n* = 7; VPA = 27.8 ± 5.5 s, *n* = 8; two-tailed *t*-test, *t*_13_ = -3.34, *p* < 0.01, **Figure 1A**), thereby indicating that in our experimental condition, prenatal VPA exposure increases anxiety-like behavior.

We also tested the effect of prenatal VPA exposure on anxiety-like behaviors using the EPM test. We found that prenatally VPA-exposed rats spent significantly less time on the open arms compared with controls (Controls = 125 ± 9.6 s, *n* = 10; VPA = 82 ± 10.9, *n* = 7; *t*_15_ = 2.93, *p* < 0.05, **Figure 1B**). In contrast, prenatal VPA exposure did not significantly alter the numbers of entries into the open (Control = 12.1 ± 0.7; VPA = 11.7 ± 1.1; *t*_15_ = 0.31, *p* = 0.76. **Figure 1C**) or the closed arms (Control = 9.5 ± 0.7; VPA = 8.7 ± 0.6; *t*_15_ = 0.75, *p* = 0.47, **Figure 1D**), indicating no significant effect on the rat locomotor activity in the EPM. Collectively, these results show that prenatal VPA exposure increases anxiety-like behavior in the EPM and that this anxiety phenotype is not associated with altered locomotor activity.

### Prenatal VPA Exposure Enhances the Activity of Putative DRn 5-HT Neurons

**FIGURE 2:**
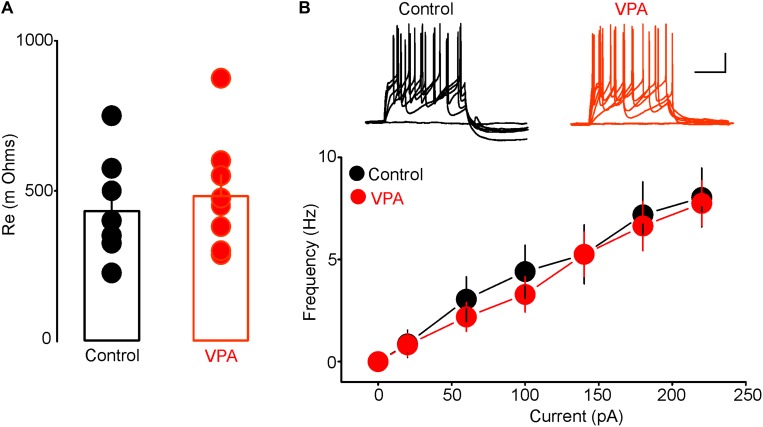
Prenatal VPA exposure has no effect on the intrinsic excitability of dorsal raphe nucleus (DRn) 5-HT neurons. **(A)** Prenatal VPA exposure does not alter input resistance of DRn 5-HT neurons. **(B)** Prenatal VPA exposure does not alter the evoked firing activity of DRn 5-HT neurons. Upper panel illustrates the responses (number of action potentials) of DRn 5-HT neurons to depolarizing current pulses obtained in slices from control (left traces) and VPA exposed rats (right traces). Lower panel depicts the average input-output curves of responses obtained in control (●, *n* = 8 cells) and VPA rats (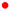, *n* = 8 cells). Data are presented as Mean ± SEM. Scale bars for the upper traces: 25 mV (vertical), 200 ms (horizontal).

To determine whether the increased anxiety-like behaviors in rats with prenatal VPA exposure were associated with alterations in excitability of putative DRn 5-HT neurons, we performed conventional *ex vivo* whole-cell recordings and assessed the impact of prenatal VPA exposure on the resting membrane potential and intrinsic excitability of these neurons. As reported previously (Vandermaelen and Aghajanian, 1983; Williams et al., 1988; Haj-Dahmane, 2001), in slices from control rats, putative DRn 5-HT neurons were quiescent and exhibited a hyperpolarized resting membrane potential (Control: -64.54 ± 2.8 mV, *n* = 16 cells). Remarkably, in slices from VPA exposed rats, all putative DRn 5-HT neurons recorded were depolarized and exhibited spontaneously firing activity (Frequency = 3.2 ± 1.5 Hz; *n* = 18 cells). These results indicate that prenatal VPA exposure increases the activity of putative DRn 5-HT neurons. In order to determine the cellular mechanisms underlying this effect, we assessed the impact of prenatal VPA exposure on the membrane input resistance and evoked firing activity of DRn 5-HT neurons. Our results showed that prenatal VPA exposure did not significantly alter the membrane resistance (Control = 481 ± 70 mΩ, *n* = 8 cells; VPA = 431 ± 60 mΩ, *n* = 8 cells, *p* > 0.05, **Figure 2A**) or evoked activity of DRn 5-HT neurons (**Figure 2B**). Taken together, these results indicate that the increased activity of DRn 5-HT neurons observed in rats with prenatal VPA exposure is unlikely to be mediated by alterations in their intrinsic excitability, but most likely by a persistent change in excitatory and/or inhibitory inputs.

### Prenatal VPA Exposure Enhances Glutamatergic Synaptic Transmission in the DRn

**FIGURE 3:**
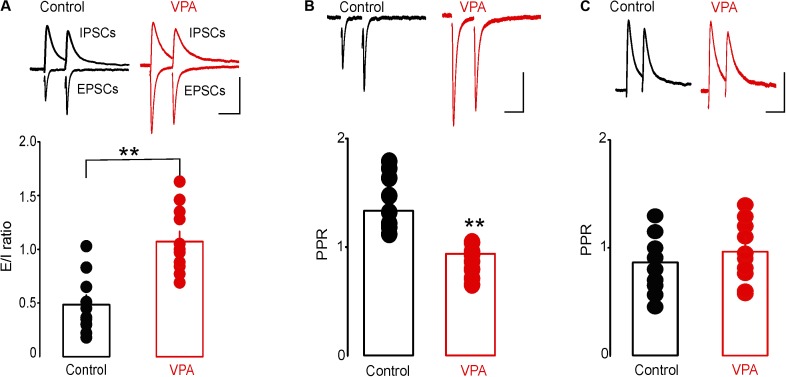
Prenatal VPA exposure increases the synaptic excitation/inhibition (E/I) ratio by enhancing glutamatergic synaptic transmission. **(A)** Prenatal VPA exposure increases the synaptic E/I ratio. Upper panel depicts superimposed excitatory/inhibitory postsynaptic current (EPSC/IPSC) traces recorded from dorsal raphe nucleus 5-HT neurons in control (left traces) and VPA rats (right traces). Lower graph shows the average E/I ratio obtained in control and VPA rats. Note that prenatal VPA exposure significantly increases the E/I ratio (control: *n* = 10 cells; VPA: *n* = 11 cells, ^∗∗^*p* < 0.01). **(B)** Prenatal VPA exposure increases the probability of glutamate release. Upper panel illustrates superimposed EPSC traces induced by pairs of stimuli at 30 ms inter-stimulus interval (ISI) and recorded in slices from control (left traces) and VPA rats (right traces). Lower graph illustrates the average paired pulse ratios (PPR = EPSC2/EPSC1) obtained at 30 ms ISI in control (●, *n* = 10 cells) and VPA rats (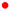, *n* = 9 cells). Prenatal VPA exposure significantly reduces the PPRs of EPSCs at ISI = 30 ms (^∗∗^*p* < 0.01). **(C)** Prenatal VPA exposure has no effect on the probability of GABA release. Upper graph illustrates superimposed pairs of GABA_A_-IPSCs recorded at 30 ms ISI in slices from control (left traces) and VPA rats (right traces). Lower graph is a summary of the average PPRs (IPSC2/IPSC1) of GABA-IPSCs obtained at 30 ms ISI in control (*n* = 6 cells) and VPA rats (*n* = 6 cells). VPA exposure has no effect on the PPR of GABA-IPSCs at ISI = 30 ms (*p* > 0.05). Data are presented as Mean ± SEM. Scale bars for the upper traces: 100 pA (vertical), 20 ms (horizontal).

To test the effect of prenatal VPA exposure on excitatory and inhibitory synaptic transmission onto putative DRn 5-HT neurons, we first assessed the E/I ratio, a measure of overall synaptic network activity in slices from control and VPA exposed rats. We found that prenatal VPA exposure significantly increased the E/I ratio (Control: E/I = 0.48 ± 0.08, *n* = 10 cells; VPA: E/I = 1.07 ± 0.09, *n* = 11 cells, *p* < 0.01, **Figure 3A**). We next examined the effects of prenatal VPA exposure on the probability of glutamate and GABA release, assessed by PPRs of EPSCs and GABA_A_-IPSCs, respectively. The results of these experiments showed that prenatal VPA exposure significantly reduced the PPRs of EPSCs determined at ISI = 30 and 50 ms (Control: PPR (30 ms) = 1.28 ± 0.09, *n* = 10 cells; VPA: PPR (30 ms) = 1.01 ± 0.04, *n* = 11 cells, *p* < 0.01, **Figure 3B**). In contrast, prenatal VPA exposure did not significantly affect the PPRs of GABA_A_-IPSCs (Control: PPR (30 ms) = 0.85 ± 0.09, *n* = 6 cells; VPA: PPR (30 ms) = 0.92 ± 0.14, *n* = 6 cells, *p* > 0.05, **Figure 3C**). Collectively, these results indicate that prenatal VPA exposure potentiates the probability of glutamate but not GABA release, which contributes to the increased E/I ratio in DRn 5-HT neurons.

**FIGURE 4:**
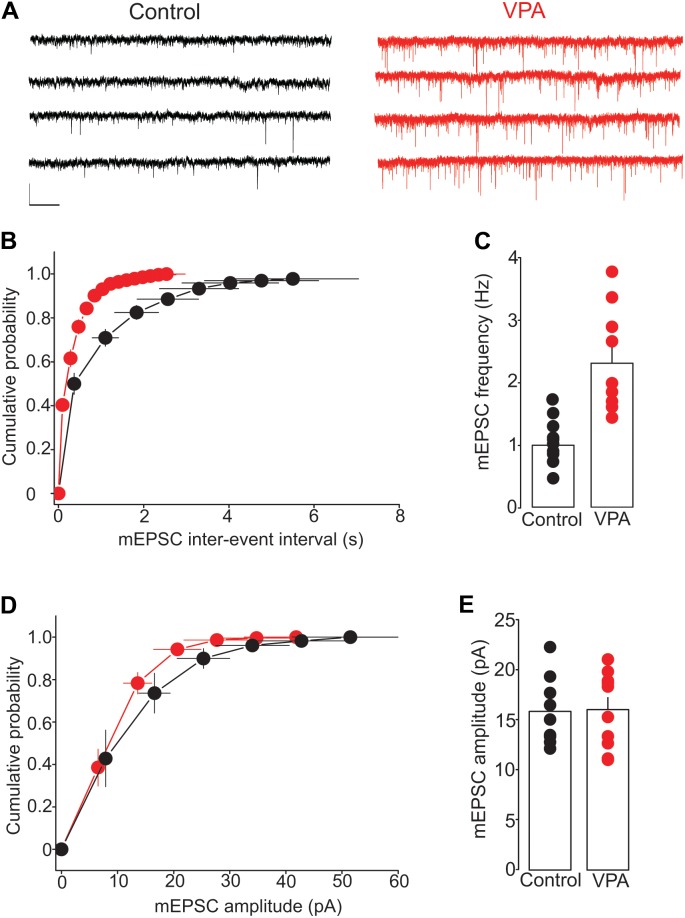
Prenatal VPA exposure increases the frequency but not the amplitude of miniature excitatory postsynaptic currents (mEPSCs). **(A)** Illustrates current traces recorded from dorsal raphe nucleus 5-HT neurons in slices from control (left traces) and VPA rats (right traces). **(B)** Represents the average cumulative distribution of mEPSC inter-event intervals obtained in control (●, *n* = 8 cells) and VPA rats (**●**, *n* = 9 cells). Prenatal VPA exposure induces a significant leftward shift of the cumulative distribution of the inter-event intervals of mEPSCs (*p* < 0.05, Kolmogorov–Smirnov test). **(C)** Depicts the average mEPSC frequencies obtained in control and VPA rats, which indicates a significant increase in mEPSC frequency in VPA rats compared with controls (^∗∗^*p* < 0.01). **(D)** Illustrates the average cumulative distribution of mEPSC amplitudes recorded in control and VPA rats. **(E)** Represents the average amplitudes of mEPSCs recorded in control and VPA rats. Note that prenatal VPA exposure has no significant effect on the cumulative distribution (*p* > 0.05, Kolmogorov–Smirnov test) of or the average mEPSC amplitude (*p* > 0.05). Data are presented as Mean ± SEM. Scale bars in (A): 10 pA (vertical), 1 s (horizontal).

To further test the effect of prenatal VPA exposure on glutamate release, we examined the frequency and amplitude of mEPSCs, a measure of quantal glutamate release. The mEPSCs were recorded from putative DRn 5-HT neurons in slices from control and VPA exposed rats. The results of this experiment showed that prenatal VPA exposure profoundly increased the frequency of mEPSCs (**Figure 4A**), which induced a significant leftward shift of the cumulative distribution of mEPSC inter-event interval (Control: *n* = 8 cells; VPA: *n* = 9 cells, *p* < 0.05, K–S test, **Figure 4B**). Indeed, the average mEPCS frequency was increased from 0.98 ± 0.1 Hz in controls to 2.23 ± 0.25 Hz in VPA exposed rats (*p* < 0.05, **Figure 4C**). In contrast, prenatal VPA exposure did not significantly alter the cumulative distribution (**Figure 4D**) of or the average mEPSC amplitude (Control = 15.9 ± 1.1 pA; VPA = 16.1 ± 1.2 pA, *p* > 0.05, **Figure 4E**). Taken together, these results indicate that prenatal VPA exposure persistently increases glutamate release at glutamatergic synapses onto DRn 5-HT neurons.

### Prenatal VPA Exposure Occludes the tLTP of Glutamatergic Synapses in the DRn

**FIGURE 5:**
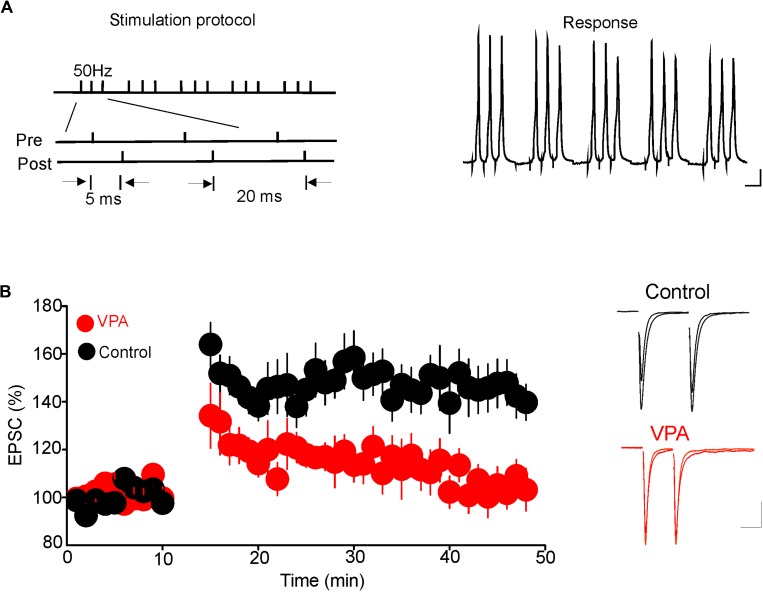
Prenatal VPA exposure occludes the spike-timing-dependent long-term potentiation (tLTP) of glutamatergic synapses in the dorsal raphe nucleus (DRn). **(A)** Depicts the stimulation protocol (left traces) and the responses (right traces) during the tLTP induction. Scale bars for the traces: 25 mV (vertical), 10 ms (horizontal). **(B)** Lower graph illustrates the time course of the tLTP in DRn slices from control (●) and VPA rats (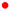). Note that pairing presynaptic stimulation with back-propagating action potentials induces a robust tLTP in control (140.5 ± 5.6 % of baseline, *n* = 10 cells, *p* < 0.01 versus baseline), but not in VPA rats (105.4 ± 3.6 % of baseline, *n* = 10 cells, *p* > 0.05 versus baseline). Right traces are superimposed excitatory postsynaptic currents recorded pre- and post-application of the tLTP induction protocol in slices from control (upper traces) and VPA rats (lower traces). Data are presented as Mean ± SEM. Scale bars for the traces: 50 pA (vertical), 20 ms (horizontal).

Results from a previous study have shown that glutamatergic synapses onto DRn 5-HT neurons undergo tLTP. Thus, pairing presynaptic stimulation with postsynaptic action potentials elicits tLTP of AMPR-EPSCs. This form of synaptic plasticity is mediated by an increase in glutamate release (Haj-Dahmane et al., 2017). Because prenatal VPA exposure persistently increases the probability of glutamate release, we examined whether this effect could occlude the tLTP in DRn 5-HT neurons. To that end, we assessed the impact of prenatal VPA exposure on the induction and magnitude of the tLTP. We found that the pairing protocol, which reliably induced tLTP in DRn 5-HT neurons of control rats, failed to induce tLTP in prenatally VPA-exposed rats (tLTP Control = 145.1 ± 10% of baseline, *n* = 10 cells; tLTP VPA = 104.8 ± 7.3% of baseline, *n* = 10 cells, *p* < 0.05, **Figure 5**). These results suggest that the persistent increase in glutamate release induced by prenatal VPA exposure occludes the tLTP.

## Discussion

Abnormal functions of the central 5-HT system are involved in several neurodevelopmental disorders including ASD. However, it remains unknown whether the function of DRn 5-HT neurons is impaired in ASD. In the present study, we show that increased anxiety and stereotypy in the VPA rat model of ASD are associated with an enhanced firing activity of putative DRn 5-HT neurons. Importantly, our results also reveal that this effect could be attributed to a persistent potentiation of glutamatergic synaptic transmission. As such, these results identify a potential cellular mechanism by which prenatal VPA exposure alters the function of the 5-HT system, which may underlie the behavioral phenotypes observed in the VPA model of ASD. The finding also provides potential cellular mechanisms mediating the pathophysiology of ASD.

Prenatal VPA exposure is a well-established environmental risk factor for the development of ASD (Christensen et al., 2013; Roullet et al., 2013; Ornoy et al., 2015). Results from numerous studies in mice and rats have shown that a single injection of VPA around embryonic day 12.5 to pregnant dams elicits ASD-like phenotypes, which include impaired social and communicative deficits and increased stereotypy (Favre et al., 2013; Roullet et al., 2013; Nicolini and Fahnestock, 2018). In addition to these core symptoms, prenatal VPA exposure also induces alterations in stress homeostasis and increases anxiety-like behaviors (Banerjee et al., 2014; Olexová et al., 2016). Consistent with these observations, results from the present study show that prenatal VPA exposure increases anxiety-like behaviors. Importantly, the behavioral phenotype is associated with physiological alterations in the DRn 5-HT system.

Previous studies examining the impact of prenatal VPA exposure on the central 5-HT system have largely focused on the effects of VPA on the early development of 5-HT neurons (Narita et al., 2002; Miyazaki et al., 2005; Tsujino et al., 2007). Generally, these studies have shown that, when administered on embryonic days 9 – 12 (neural plate stage), VPA irreversibly alters the differentiation and migration of 5-HT neurons to the DRn (Narita et al., 2002; Tsujino et al., 2007). In addition, prenatal VPA exposure has been shown to increase 5-HT levels in the forebrain areas (Narita et al., 2002; Tsujino et al., 2007), suggesting an enhanced 5-HT neurotransmission in VPA exposed rats. Results from the present study reveal for the first time, that prenatal VPA exposure persistently increases the firing activity of DRn 5-HT neurons. This observation provides a cellular mechanism by which prenatal VPA exposure enhances central 5-HT transmission. Importantly, because hyperserotonemia is thought to mediate increased anxiety, our findings provide a potential neuronal mechanism for behavioral dysfunctions in ASD.

The results from the present study show that the increased firing activity of DRn 5-HT neurons in rats with prenatal VPA exposure may be mediated by alterations in their synaptic inputs. Indeed, we have shown that prenatal VPA exposure persistently increases the ratio of synaptic E/I in DRn 5-HT neurons. Interestingly, an increase in synaptic E/I ratio has also been reported in other brain areas of prenatally VPA exposed rats (Kim et al., 2013; Lin et al., 2013) and numerous genetic models of ASD (Rubenstein and Merzenich, 2003; Dani et al., 2005; Lee et al., 2015). Collectively, these studies indicate that an imbalance of synaptic E/I is a common effect of both environmental and genetic risk factors for ASD, which could play a major role in the pathophysiology of ASD (Yizhar et al., 2011; Lee et al., 2017).

The results from the present study show that the increased E/I ratio in DRn 5-HT neurons of VPA exposed rats is mainly mediated by a persistent potentiation of glutamate release. This conclusion is supported by the observation that prenatal VPA exposure decreases the PPR of EPSCs and increases the frequency of mEPSCs, but has no significant effect on GABAergic synaptic transmission. These results are consistent with previous studies reporting increased glutamate release in other brain areas of VPA exposed rats (Markram et al., 2008; Lin et al., 2013). Such results support the notion that an enhanced glutamatergic transmission is a common mechanism underlying the synaptic E/I imbalance observed in the VPA model of ASD. In contrast, studies using various genetic models of ASD have reported that alterations in both glutamate and GABA concentrations and/or synaptic transmission contribute to the imbalance of synaptic E/I (Lee et al., 2017). For example, BTBR mice with ASD behavioral phenotypes have both increased glutamate and decreased GABA concentrations in the prefrontal cortex (PFC) and amygdala (Bove et al., 2018).

Prenatal VPA administration has also been shown to alter synaptic plasticity. Indeed, VPA exposed rats exhibit an enhanced NMDA receptor function (Rinaldi et al., 2007; Kang and Kim, 2015) and NMDA-mediated LTP of glutamatergic synapses in the lateral amygdala (Markram et al., 2008; Lin et al., 2013) and the medial PFC (Rinaldi et al., 2008; Sui and Chen, 2012). We show that in DRn 5-HT neurons, while glutamatergic transmission is enhanced in VPA exposed rats, the tLTP of glutamatergic synapses onto 5-HT neurons is impaired. Because the tLTP in DRn 5-HT neurons is mediated by presynaptic increase in glutamate release and prenatal VPA exposure persistently increases glutamate release, the inhibition of tLTP observed in VPA exposed rats is most likely mediated by an occlusion effect. These observations suggest that the glutamatergic synaptic strength is maximized and the normal function of synaptic homeostasis is impaired in DRn 5-HT neurons of VPA rats. Such an effect could further contribute to behavioral deficits associated with 5-HT function (Daly et al., 2014).

We have previously shown that the tLTP of glutamatergic synapses onto DRn 5-HT neurons is mediated by the activation of nitric oxide (NO) and the protein kinase G signaling pathways (Haj-Dahmane et al., 2017). Interestingly, results from numerous studies have shown that prenatal VPA exposure promotes oxidative stress and enhances the formation of nitric oxide (Frustaci et al., 2012; Mario Tiboni and Ponzano, 2014; Yui et al., 2016). These effects are mediated by an increased function and expression of neuronal nitric oxide synthase (nNOS) (Mario Tiboni and Ponzano, 2014). Thus, it is tempting to speculate that the increased glutamate release and occlusion of the tLTP in DRn 5-HT neurons of VPA exposed rats could be mediated by enhanced NO signaling. However, future studies are required to directly confirm this hypothesis.

Taken together, results from the present study show that a major phenotype of ASD, increased anxiety-like behaviors, is observed after prenatal VPA exposure, a well-characterized non-genetic model of ASD. Importantly, we show increased DRn 5-HT firing caused by enhanced glutamatergic neurotransmission in VPA exposed rats. This effect is accompanied by impaired plasticity in glutamatergic synapses. It is well established that the DRn 5-HT system plays an important role in mediating anxiety-like behaviors (Iversen, 1984; Graeff et al., 1996). Therefore, our results provide potential cellular mechanisms for these behaviors associated with hyperserotonemia in individuals with ASD.

## Author Contributions

RW conducted animal breeding and performed the behavioral experiments. KH and R-YS conducted prenatal animal treatment, participated in the experimental design, and coordinated the study. SH-D directed the project and performed the electrophysiological experiments. SH-D, RW, and R-YS wrote the manuscript.

## Conflict of Interest Statement

The authors declare that the research was conducted in the absence of any commercial or financial relationships that could be construed as a potential conflict of interest.
